# Serosurvey and Associated Risk Factors for Bovine Viral Diarrhea Virus Infection in Dromedary Camels in Egypt

**DOI:** 10.1155/2024/3188539

**Published:** 2024-02-10

**Authors:** Abdelfattah Selim, Mohamed Marzok, Abdelhamed Abdelhady, Hattan S. Gattan, Mohamed Salem, Mohammed Ali Al-Hammadi

**Affiliations:** ^1^Department of Animal Medicine (Infectious Diseases), Faculty of Veterinary Medicine, Benha University, Toukh 13736, Egypt; ^2^Department of Clinical Sciences, College of Veterinary Medicine, King Faisal University, Al-Ahsa 31982, Saudi Arabia; ^3^Department of Surgery, Faculty of Veterinary Medicine, Kafr El Sheikh University, Kafr El Sheikh, Egypt; ^4^Department of Parasitology and Animal Diseases, National Research Center, Giza, Egypt; ^5^Department of Medical Laboratory Sciences, Faculty of Applied Medical Sciences, King Abdulaziz University, Jeddah, Saudi Arabia; ^6^Special Infectious Agents Unit, King Fahad Medical Research Center, King AbdulAziz University, Jeddah, Saudi Arabia; ^7^Department of Microbiology, College of Veterinary Medicine, King Faisal University, Al-Ahsa, Saudi Arabia; ^8^Department of Medicine and Infectious Diseases, Faculty of Veterinary Medicine, Cairo University, Cairo 12613, Egypt

## Abstract

Bovine viral diarrhea (BVD) is a disease that affects ruminants globally, including camels, and causing significant financial losses. The epidemiology of BVD in camels in Egypt are not well understood. Thus, this study aimed to determine the prevalence of anti-BVD virus (BVDV) antibodies in camels and identify the potential variables associated with the infection. A total of 400 camel sera from three Egyptian governorates were examined using commercial ELISA kit. The total seroprevalence was 4.8% in examined camels and the BVDV seropositivity were more prevalent in camels from Giza governorate. The highest seropositivity was found in aged camels more than 8 years (OR = 8.62, 95%CI: 1.03–71.87), camels from herd size less than 30 (OR = 4.20, 95%CI: 0.89–19.78), previously aborted animals (OR = 5.98, 95%CI: 2.12–16.92), and in animals kept in contact with sheep or goats (OR = 7.48, 95%CI: 2.56–21.86). Consequently, the camels may be a significant source of BVD infection for other ruminant animals in the same herd due to their susceptibility to the viral infection.

## 1. Introduction

Camels are ship of desert which are present mainly in middle east and parts of northern Africa. Camel has been used as a useful multipurpose animal throughout history for transportation, milk production, wool production, meat production, and other purposes [1, 2]. Nowadays, camel breeding is mostly focused on producing meat or tourists, and as a result of technological and industrial improvements, this animal has lost some of its significance [3].

According to literatures, camels are vulnerable to several diseases that impact other animal species, including bluetongue, brucellosis, toxoplasmosis, rift valley fever, bovine herpesvirus-1 (BoHV-1), *Mycobacterium avium* subsp. *paratuberculosis*, and coxiellosis [4–9].

Bovine viral diarrhea virus (BVDV) is a member of the *Pestivirus* genus within the *Flaviviridae* family, that causes bovine viral diarrhea (BVD) disease. BVDV have two genotypes, BVDV-1 and BVDV-2, each of which possesses cytopathic and noncytopathic biotypes, which effect on the pathogenesis and epidemiology of BVDV in host including cattle and camels [10, 11].

BVD is a global problem that causes substantial economic losses since it causes abortion, decrease the conception rate and milk production, and prolongs calving intervals [12, 13]. BVD is a widespread disease in cattle, but it is an emerging disease in both new- and old-world camels that can be infected by sharing pasture with cattle [14, 15]. Moreover, the disease causes diarrhea, reproductive failure, and respiratory symptoms in camels [16, 17]. The prevalence of BVD in camels varied between different countries, it was 3.4% in Somalia [18], but it was more prevalent among camels in Sudan 16% [19], in Iran 19.7% [20], and Egypt 52.2% [21].

In Egypt, BVD is prevalent among domestic ruminants such as sheep, goats, cattle, and buffaloes [22, 23]; however, few studies have reported the BVDV infection in camels but in few governorates without assessment of risk factors for the infection.

While there are multiple BVDV vaccinations available for use in cattle, no vaccine has been authorized for use in camelids as of yet. Although vaccinations cannot stop infection, they can decrease the appearance of clinical signs [24]. It may be possible to reduce the frequency of infections by keeping a sealed herd, enforcing strict biosecurity requirements for all animals entering the herd, and routinely examining open herds [14].

Therefore, this study aimed to determine the prevalence of BVDV infection and the potential risk factors associated with the infection in camels from three governorates in Egypt.

## 2. Materials and Methods

### 2.1. Ethical Statement

All methods and procedures including in this study were approved by ethical committee of Benha University and followed all rules and instructions of committee. The entire study procedure was conducted in accordance with the ARRIVE standards.

### 2.2. Study Areas

**Figure 1:**
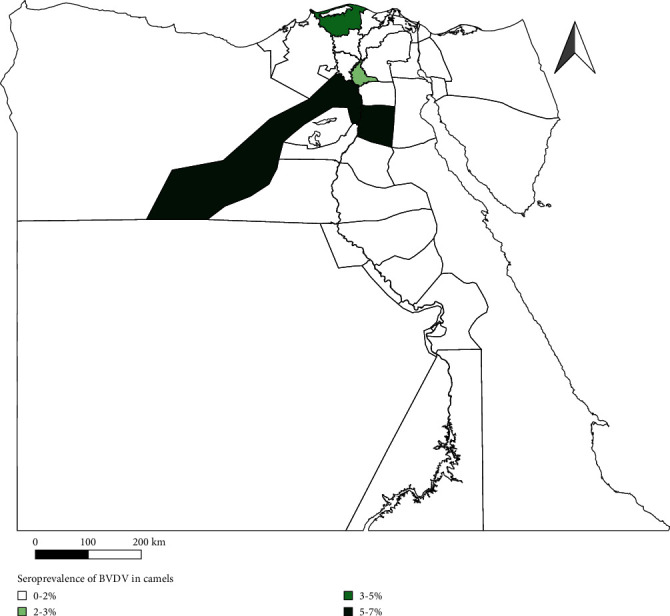
Prevalence of anti-BVDV antibodies in camels from different locations (MAP generated using QGIS software).

A cross-sectional study was conducted during the period from January 2022 to December 2022 in three governorates (Giza, Kafr ElSheikh, and Marsa Matrouh) in Egypt (Figure 1). These three governorates were selected because camel is one of the most common species and using as source of meat, breeding mainly in Kafr ElSheikh and Marsa Matrouh, while it has significant role in ancient tourist places in Giza. Giza situated opposite to central of Cairo between 29.9870°N latitude and 31.2118°E longitude and its climate has hot and dry in summer with cool winter. Kafr ElSheikh situated at northern of Egypt within the Nile Delta and along western side of Nile. In addition, Kafr ElSheikh has lengthy, hot, humid, and clear summer and mild, windy, and dry winter. Marsa Matrouh is located in northwestern Egypt, on the Mediterranean coast, in the Libyan desert. The yearly average temperature of Marsa Matruh is 20.0°C, while the average yearly precipitation is 63 mm.

### 2.3. Sample Size and Sampling

The following formula was used to determine the sample size, according to Thrusfield [25].

(1) $$\begin{array}{c} N=Z^{2}\times P\left(1-P\right)/d^{2} \end{array}$$

where *n* is the sample number, *P* is the previous prevalence rate which was 2.18% [26] for BVD in camels, with 95% confidence interval, and *d* is absolute error (5%).

A total of 400 serum samples were collected randomly from asymptomatic camels raising in the three studied governorates. Blood samples were collected based on a random representative number of herds which was chosen initially, followed by a random representative number of animals each herd. A total of 32 camel herds were examined in the studied areas and classified to two sizes (≤30 or >30).

These animals did not receive vaccination against BVD and had no symptomatic clinical signs for BVD. The animal was carefully restrained before a 10 mL blood sample was drawn from the jugular vein using a vacutainer tube. The sera were separated by centrifugation at 1,500x *g* for 10 min and stored at −20°C for subsequent serological investigation.

### 2.4. Serological Analysis

Commercial ELISA kits (IDvet, Innovative Diagnostics, Grabels, France) for detection of BVDV p80 antibody in serum of examined camels was used, following the manufacturer's instructions. This kit has 100% sensitivity and specificity to identify the presence of circulating antibodies in the serum [17]. The absorbance of the sample was measured at 450 nm using ELISA microplate reader. The percent competition of each sample (*S*/*N*%) was calculated using the formula specified in the test procedure.

(2) $$\begin{array}{c} \text{S/N}\%={\text{OD}}_{\text{sample}}/{\text{OD}}_{\text{NC}}\times 100 \end{array}$$

When the *S*/*N*% was 40%, 40–50%, or >50%, the result was evaluated as positive, uncertain, or negative.

### 2.5. Questionnaire

At time of sampling, data of each examined animals were collected using epidemiological questionnaire to analyze the risk factors associated with BVDV infection in camels. The following variables were examined and their corresponding categories are as follows: sex (male/female), age (6 months ≤4 years, 4–8 years, >8 years), herd size (≤30/>30), contact with sheep and goats (yes/no), and history of abortion (yes/no).

### 2.6. Statistical Analysis

IBM SPSS program for Windows, version 24 (Armonk, USA: IBM) was used for all statistical analyses. Univariable regression method was used to assess each regression coefficient's statistical significance. Statistical significance was set at *P* < 0.05. The relationship between subject variables and BVDV infection was assessed using multivariable logistic regression methods [27–29]. A variable was considered to be part of the multivariable analysis if its *P* value in the univariable analysis was ≤0.25. The Hosmer and Lemeshow test was used to validate the final model fit, and correlation analysis was used to check for collinearity between the independent variables. Correlation coefficient was >0.9 for variables with substantial collinearity.

## 3. Results

Based on animal level, 19 (4.8%, 95% CI: 3.06–7.3) were positive for BVDV antibodies. In addition, 27 herds had at least one positive sample and the seroprevalence rate according to herd level was 64.3%, 95%CI: 68.25–93.14 (Table 1).

The results revealed that no significant (*P*  > 0.05) effect for sex and locality on prevalence of BVDV in camel but BVDV seropositivity in camels was significantly influenced (*P*  < 0.05) by age, herd size, history of abortion, and contact with sheep and goats. The highest seroprevalence was seen in camels above the age of 8 years (8.9%, 95% CI: 5.16–14.9), particularly in herds of ≤30 animals (6.5%, 95% CI: 4.12–10.22), as shown in Table 2.

Camels' seropositivity for BVDV was highly correlated with their history of abortion or contact with sheep and goats. The seroprevalence of BVDV rose significantly in camels with a history of abortion (13.8%, 95%CI: 7.46–24.27) and in camels living in contact with sheep and goats (10.8%, 95%CI: 6.44–17.65), Table 2.

Moreover, Camels older than 8 years (OR = 8.62, 95%CI: 1.03–71.87) and those in a herd with ≤30 animals (OR = 4.20, 95%CI: 0.89–19.78) had nine and four times higher odds of being seropositive for BVDV, respectively, according to the multivariable analysis (Table 3). In addition, camels with a history of abortion (OR = 5.98, 95%CI: 2.12–16.92) and those had contact with sheep and goats (OR = 7.48, 95%CI: 2.56–21.86) had a higher probability to be seropositive for BVDV compared to other animals, Table 3.

## 4. Discussion

For many years, Egypt has imported camels from Sudan for usage in drafting work, meat production, or as mounts for the border police. Camels are prone to several viral illnesses, and they contribute significantly to the etiology of these illnesses as well as the multiplication of some viruses [5, 14, 16]. This study aims to evaluate the prevalence of BVDV and its related risk factors because the epidemiological knowledge regarding BVDV in camels in Egypt is scarce.

In this study, the overall prevalence of antibodies against BVDV in camels was 4.8%. In addition, vaccination against BVDV is not used in Egypt, indicating that the prevalence of these antibodies is due to natural infection.

The prevalence reported in this study was consistent with the published rate in Iranian camels (4.7%) [30], although it was lower than the rates reported in Turkey (16.8%) [31], Egypt (27.2%) [32], and Iran (19.7%) [20].

However, the prevalence of BVDV antibodies in camels globally ranged from 0% to 84.6% [16, 33–37]. Furthermore, in certain countries, such as China and Sudan, detection of BVDV has been reported in clinical samples from camels [38]. Gao et al. [35] discovered a significant level of diversity among BVDV-1 strains isolated from camels. According to recent publications, BVDV p80 ELISA and serum neutralization tests revealed significant seroprevalence rates ranging from 37% to 58.7% in dromedary camels in the Aydin province of Turkey [31, 39]. However, ELISA did not reveal any BVDV antigen positive [40].

There are some countries with higher prevalence of BVDV infection among camels, such as Sudan and Saudi Arabia, which breed camels mostly for meat and milk production, and usually in mixed herds with other livestock [41, 42]. These differences in seroprevalence might be attributed to several variables such as density of population, strategies of disease prevention, managemental factors, and animal age [43].

This study found that the prevalence of BVDV infection rose with the age of the camels. Camel over 8 years old had the highest precalence (8.9%) compared to camels under 4 years old (1.4%). This result is consistent with prior findings of Raoofi et al. [20] and Saidi et al. [16].

This finding might be related to older camels being more exposed to BVDV and, as a result, having a greater chance of identifying BVDV seropositivity [35, 40].

When compared to camels raised alone and in large herds, the prevalence of BVDV infection was considerably greater in camels in small herds that raised together with small ruminants. A similar results were reached by Ataseven et al. [31] and Tesfaye et al. [17]. A number of previous studies have been reported that aggregation with other ruminants in pasture, size of herd, age of animals, and introduction of new animals from uncontrolled markets without efficient examination increased the possibility and chance for transmission of many of viral diseases [44–46]. Moreover, seroconversion in camels most likely occurred in case of direct contact with infected ruminants within the same pastures or indirectly via contact with contaminated fomites [31]. Moreover, even if these ruminants species are not in direct contact, persistently infected animals spread BVDV via contaminating the environment or sharing equipment [47]. In countries with high prevalence of BVDV infection in camels, such as Sudan, Egypt, and Saudi Arabia, camel breeding is primarily for economic reasons, such as meat and milk production. In addition, herds are typically mixed with other ruminants and interspecies transmission has also been recorded [16, 48].

In the line of previous results of Tesfaye et al. [17], camels with history of abortion were more likely to get the infection than others. This discovery supports the hypothesis that the virus has a high preference for the reproductive organs of affected animals, altering ovarian function and ultimately resulting in infertility [49]. However, more research is needed to clarify the role that BVD plays in reproductive failures in pastoral settings.

The results of this study have some limitations due to the most of examined samples were collected randomly from three governorates which have highest camel population.

## 5. Conclusion

This study found subclinical BVDV infection in the camel population in the governorates under the study. Because there is no vaccine program against this disease in Egypt, the data point to natural exposure. The multivariable logistic regression showed that age, smaller herd, contact with other species like sheep and goats, or history of abortion are identified as potential risk factors for BVDV infection in camels. Therefore, the wide epidemiological survey should be implemented to determine the distribution of BVDV among camels and to apply reliable control program to prevent spread of infection among ruminants.

## Figures and Tables

**Table 1:** Animal and herd level seroprevalence of BVDV in camels based on ELISA.

Sampling area	Herd level				Animal level			
	Tested	Positive	Prevalence (%)	95%CI	Tested	Positive	Prevalence (%)	95%CI
Kafr ElSheikh	12	10	83.3	55.19–95.3	130	6	4.6	2.14–9.71
Giza	6	4	66.7	30–90.32	150	10	6.7	3.66–11.84
Marsa Matrouh	14	13	92.9	68.53–98.73	120	3	2.5	0.85–7.09
Total	32	27	64.3	68.25–93.14	400	19	4.8	3.06–7.3

**Table 2:** Seroprevalence of BVD antibodies in camels from Egypt.

Variable	Total tested camels	No. of positive	No. of negative	% of positive	95%CI	Statistic
Locality						
Kafr ElSheikh	130	6	124	4.6	2.14–9.71	*χ*2 = 2.566 df = 2 *P*=0.277
Giza	150	10	140	6.7	3.66–11.84	
Marsa Matrouh	120	3	117	2.5	0.85–7.09	
Sex						
Male	80	2	78	2.5	0.69–8.66	*χ*2 = 1.119 df = 1 *P*=0.290
Female	320	17	303	5.3	3.34–8.34	
Age	—	—	0	—	—	—
6 months to ≤4 years	71	1	70	1.4	0.25–7.56	*χ*2 = 7.994 df = 2 *P*=0.018 ^*∗*^
4–8 years	193	6	187	3.1	1.43–6.62	
>8 years	135	12	123	8.9	5.16–14.9	
	399	19	380	4.8	—	—
Herd size						
≤30	260	17	243	6.5	4.12–10.22	*χ*2 = 5.252 df = 1 *P*=0.022 ^*∗*^
>30	140	2	138	1.4	0.39–5.06	
Contact with sheep and goats	—	—	0	—	—	—
Yes	120	13	107	10.8	6.44–17.65	*χ*2 = 14.002 df = 1 *P* < 0.0001 ^*∗*^
No	280	6	274	2.1	0.98–4.59	
History of abortion						
Yes	65	9	56	13.8	7.46–24.27	*χ*2 = 15.473 df = 1 *P* < 0.0001 ^*∗*^
No	255	10	245	3.9	2.14–7.07	
Total	400	19	381	4.8	3.06–7.3	—

^*∗*^*P*  < 0.05 indicates that the result is significant.

**Table 3:** Multivariable logistic regression analysis for potential risk variables associated with BVDV infection in camels.

Factor	B	S.E.	OR	95%CI for OR		*P* value
				Lower	Upper	
Age						
4–8 years	1.162	1.118	3.20	0.36	28.59	0.030
>8 years	2.154	1.082	8.62	1.03	71.87	0.046
Herd size						
≤30	1.434	0.791	4.20	0.89	19.78	0.050
Contact with sheep and goats						
Yes	2.012	0.547	7.48	2.56	21.86	<0.0001
History of abortion						
Yes	1.789	0.530	5.98	2.12	16.92	0.001

B, logistic regression coefficient; SE, standard error; OR, odds ratio; and CI, confidence interval.

## Data Availability

All data generated or analyzed during this study are included in this published article.
